# 4-Thiazolidinone derivative Les-3833 effectively inhibits viability of human melanoma cells through activating apoptotic mechanisms

**DOI:** 10.3325/cmj.2017.58.129

**Published:** 2017-04

**Authors:** Nataliya Finiuk, Nataliya Boiko, Olga Klyuchivska, Lesya Коbylinska, Iryna Kril, Borys Zimenkovsky, Roman Lesyk, Rostyslav Stoika

**Affiliations:** 1 Department of Regulation of Cell Proliferation and Apoptosis, Institute of Cell Biology, National Academy of Sciences of Ukraine, Lviv, Ukraine; 2 Department of Biochemistry, Danylo Halytsky Lviv National Medical University, Lviv, Ukraine; 3 Department of Clinical Immunology and Allergology, Danylo Halytsky Lviv National Medical University, Lviv, Ukraine; 4 Department of Pharmaceutical, Organic and Bioorganic Chemistry, Danylo Halytsky Lviv National Medical University, Lviv, Ukraine

## Abstract

**Aim:**

To evaluate cytotoxic action of 4-thiazolidinone derivative Les-3833 and study the mechanisms of its pro-apoptotic action toward human melanoma cells and human tumor cell lines of other tissue origin.

**Methods:**

The effect of Les-3833 or doxorubicin on the viability of 9 cell lines was studied using MTT assay, while human melanoma cells of WM793 line were additionally examined using light and fluorescent microscopies for evaluating cytomorphological changes. The Western-blot and flow cytometric analyses were carried out to study signaling pathways of melanoma cell cycling and death.

**Results:**

Les-3833 was the most efficient against melanoma cells. Its half maximal inhibitory concentration (IC_50_) was 0.22 μg/mL for WM793 cells and 0.3 μg/mL for SK-Mel-28 melanoma cells. For human lung A549, breast MCF-7, colon HCT116, and ovarian SKOV3 carcinoma cell lines IC_50_ was in between 2.5 to >5.0 μg/mL. Les-3833 was relatively not toxic (IC_50_ ˃ 5 μg/mL) for human embryonic kidney HEK293 cells. Results of Annexin V/PI staining of melanoma cells and activation of caspase 3, PARP, MAPK, and EndoG protein suggest apoptosis in Les-3833-treated cells. Les-3833 also induced ROS production in melanoma cells and their arrest in G_0_/G_1_ phase of cell cycle.

**Conclusion:**

Novel 4-thiazolidinone derivative Les-3833 is effective against human melanoma cells *in vitro,* and such effect is tumor specific since it is much less pronounced in human carcinoma and leukemia cells. In melanoma cells Les-3833 induces apoptosis (morphological changes and increased pro-apoptotic proteins), ROS production, and arrest in G_0_/G_1_ phase of cell cycle.

Melanoma arises from the melanin-producing skin cells - melanocytes. It exhibits high metastasis potential and poor prognosis in treated patients with a survival rate of 16.1% (1).

Since there is no effective anti-melanoma drugs available in clinics, melanoma remains as one of the most difficult for chemotherapeutic treatment (2). That is why, the immunomodulating approaches were applied. They include the application of cytokines (high-dose of interferon alfa-2b (Intron A) and interleukin-2), and of the antibodies (ipilimumab, anti-CTLA4 and anti-PD-1 antibodies). Other new strategies for melanoma treatment are based on using immune modulators, BRAF inhibitors (Vemurafenib) and MEK (mitogen-activated protein kinase) inhibitors. All these drugs are very costly, and some of them can be highly toxic and not effective (3,4). Subsequently, any success in creating novel anti-melanoma drug is a big challenge in development of effective chemotherapy for this highly malignant tumor.

Usually, the chemotherapeutic compounds impair not only tumor cells but also exhibit significant negative side effects toward non-tumor cells. In addition, drug resistance of the melanoma cells develops with high rate. Targeting cell proliferation and apoptotic pathways are principal approaches for understanding pathogenesis of most diseases including cancer. Thus, the agents capable of blocking cell cycle and inducing apoptosis of tumor cells are attractive as novel anticancer medicines (5).

4-Thiazolidinones derivatives have been used for the design of novel drugs (6,7). These substances demonstrate wide spectrum of biological effects, including antibacterial, anti-mycotic, hypoglycemic, antineoplastic, immunomodulating, and antidiabetic (8-12). Moreover, 4-thiazolidinone core possesses high capacity for chemical modifications that opens great possibilities in the development of novel derivatives. Principal approaches in modification of 4-thiazolidinone-bearing compounds are focused on the creation of new antibacterial, antiviral, anti-inflammatory, antidiabetic, and anticancer agents (12). 4-Thiazolidinones were also used for treatment of neuropathy and nephropathy (8). Such compounds induced changes in Ca^2+^ level, the mitogen-activated protein kinases (MAPK) activation, reactive oxygen species (ROS) production and endoplasmic reticulum stress (5,13).

Recent achievements in the medicinal chemistry of 4-thiazolidinones have significantly stimulated the development of studies addressed on a design of new anticancer agents (5). It was reported that products of the hybridization of thiazolidine-2,4-diones scaffolds with different bioactive molecules possessed anticancer activity (5). It was shown that novel 5-ene-4-thiazolidinones possessed a selective anti-leukemic action (14). A search for novel potent antitumor pharmaceuticals demonstrating high selectivity and low toxicity to normal cells is currently strongly prioritized (5,8,12-14).

In the present work, we evaluated novel synthetic 4-thiazolodinone derivative, the Les-3833, as a potent anti-melanoma agent, and compared its toxic action toward tumor cells of other tissue origin, as well as studied the molecular mechanisms of the pro-apoptotic action of this compound.

## Methods

### Chemical compounds

**Figure 1:**
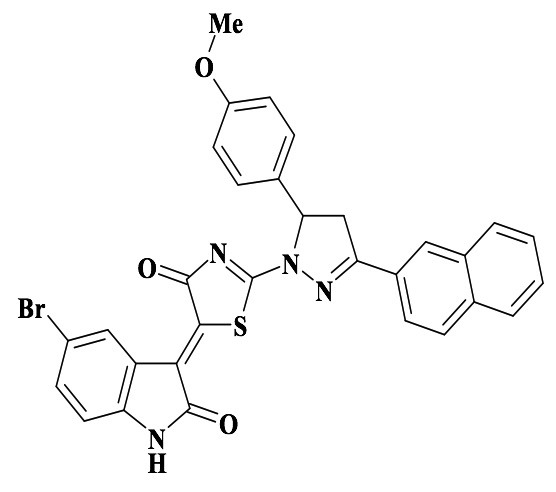
Chemical structure of Les-3833 – 5-bromo-3-{2-[5-(4-methoxyphenyl)-3-naphthalen-2-yl-4,5-dihydropyrazol-1-yl]-4-oxo-4,5-dihydro-1,3-thiazol-5-ylidene}-2,3-dihydro-1H-indol-2-one.

The heterocyclic 4-thiazolidinone derivative Les-3833 (Figure 1) belongs to the pyrazoline-thiazolidinone-isatins conjugates and was synthesized as described previously (15). Stock solution of Les-3833 (10 mM) was prepared in the dimethyl sulfoxide (DMSO, REALAB, Kyiv, Ukraine), and dissolved in cell culture medium before addition to the cell culture medium. Doxorubicin (Dox, TEVA Pharmachemie B.V., Haarlem, the Netherlands) was used, as a reference anticancer drug.

### Cell culture

Human ovarian carcinoma SKOV3 cells and human melanoma WM793 cells were received from the American type culture collection (ATCC, Minnesota, USA) and were provided by Dr O. Stasyk (Institute of Cell Biology, National Academy of Sciences of Ukraine, Lviv, Ukraine). Human melanoma SK-MEL-28 cells, human lung carcinoma A549 cells, human breast adenocarcinoma MCF-7 cells, human embryonic kidney HEK293 cells were obtained from the collection at R.E. Kavetsky Institute of Experimental Pathology, Oncology and Radiobiology (Kyiv, Ukraine). Human colon carcinoma HCT116 cells, human keratinocytes of HaCat line and human leukemia K562 cells were donated from a collection of the Institute of Cancer Research at Vienna Medical University (Vienna, Austria). Cells were grown in the RPMI-1640 or DMEM (Sigma-Aldrich, St. Louis, USA) culture media supplemented with 10% fetal bovine serum (Sigma-Aldrich, St. Louis, USA). Cells were incubated in the CO_2_-thermostate at 37°C in the atmosphere containing 5% CO_2_.

### Anti-proliferative assay

*In vitro* screening of the anticancer action of the synthetized compound Les-3833 and doxorubicin towards tumor cell was measured using the 3-(4,5-dimethylthiazol-2-yl)-2,5-diphenyl-tetrazolium bromide (MTT) test (Sigma-Aldrich, St. Louis, USA). Cells were seeded for the night in 100 μL in the 96-well plates: substrate-dependent cells were seeded at the concentration of 2000 cells/well and suspension cells - at 5000 cells/well. Then, the cells were treated with the Les-3833 and doxorubicin (none; 0.1; 0.25; 0.5; 1.0; 5.0; 10.0 μg/mL) and incubated for next 72 h. The mitochondrial dehydrogenases converted the MTT to its colored product and water insoluble MTT formazan (additionally dissolved in the DMSO) was used to determine the viable cells. Product of the reaction was determined by an Absorbance Reader BioTek ELx800 (BioTek Instruments, Inc., Winooski, VT, USA) at 620 nm wavelength.

In order to study time-dependent cytotoxicity of Les-3833 and Dox, cells of WM793 line were treated with these compounds (none; 0.1; 0.25; 0.5; 1.0; 5.0; 10.0 μg/mL) and incubated for the subsequent 6, 24, 48 and 72 h. After that, MTT assay was used to determine the viable cells.

For estimating the impact of ROS’s scavengers (N-acetyl-cysteine (NAC) and ascorbic acid (AA) (Sigma-Aldrich, St. Louis, USA)) on the cytotoxicity of Les-3833 and Dox on WM793 cells were used in a combination with cell pre-treatment for 30 min with the non-toxic concentrations of ROS scavengers (NAC was used in 1.0; 2.0 mM: AA - at 25.0; 100.0 μM) (14).

### Fluorescent microscopy

The cells of WM793 line were seeded on the glass microscopic slides in the 12-well plates. Double staining of cells with FITC-conjugated Annexin V and Propidium iodide (PI) (both from Sigma-Aldrich, St. Louis, USA) was used to identify early apoptotic events in human melanoma WM793 cells treated with Les-3833 and Dox (both used at 0.25 µg/mL concentration) for 72 h. After that, cells were washed with 1 × phosphate buffered saline (PBS), and incubated for 15 min with the Annexin V binding buffer containing 1/20 volume of FITC-conjugated Annexin V solution and PI (20.0 μg/mL) (14). The cells were analyzed under Zeiss fluorescent microscope (Carl Zeiss, Jena, Germany) using AxioImager A1 camera.

In 72 h after the addition of the tested compounds at 1 µg/mL concentration, the cells were stained with 0.2-0.5 µg/mL of the DNA-specific fluorescent dye Hoechst 33342 (Sigma-Aldrich, St. Louis, USA). The cells were also stained by the poly-specific dye Acridine orange (AO, 0.3-1.0 µg/mL, Sigma-Aldrich, St. Louis, USA). The cells were incubated for next 20-30 min and examined using a Zeiss fluorescent microscope (Carl Zeiss, Jena, Germany) using AxioImager A1 camera.

### Western-blot analysis

Proteins of cells treated with the tested compound (Dox was used in 0.25 µg/mL concentration, Les-3833 - in 0.25 and 0.5 µg/mL) were separated by the SDS/PAGE gel-electrophoresis and transferred onto a polyvinylidene difluoride (PVDF) membrane, as described (13). The antibodies for Cleaved Caspase 3 (Asp175), Cleaved PARP (Asp214), anti-phospho-ERK1/2 (phospho-p44/42, Thr 202/Tyr 204), anti-EndoG, anti-STAT3, anti-phospho-Rb (Ser 807/811), (Cell Signaling Technology, New England Biolabs GmbH, Austria), anti-Cdk2 (SantaCruzBiotechnology, Inc., Dallas, Texas, USA), anti-beta-actin monoclonal mouse AC-15 (Sigma-Aldrich, St. Louis, USA) were used. Secondary rabbit and mouse peroxidase-labeled antibodies (CellSignaling Technology, New England Biolabs GmbH, Austria) were used at working dilution of 1:5 000.

### Cell cycle analysis

To analyze cell cycle distribution, WM793 cells were seeded into 6-well plates and then treated with the Les-3833 or doxorubicin at 0.25 µg/mL for 72 h. Cells were prepared as described (14), stained with PI (5 μg/mL) and analyzed by flow cytometry (Becton Dickinson, PaloAlto, CA, USA).

### Statistical analysis

Results were presented as the mean ± standard deviation (SD). GraphPad Prism 6 was used for results analysis, charting, *t* test or two-way ANOVA test. *P* < 0.05 was set as the level of statistical significance.

## Results

### *Anticancer activity* in vitro *of 4-thiazolidinone derivative Les-3833*

**Figure 2:**
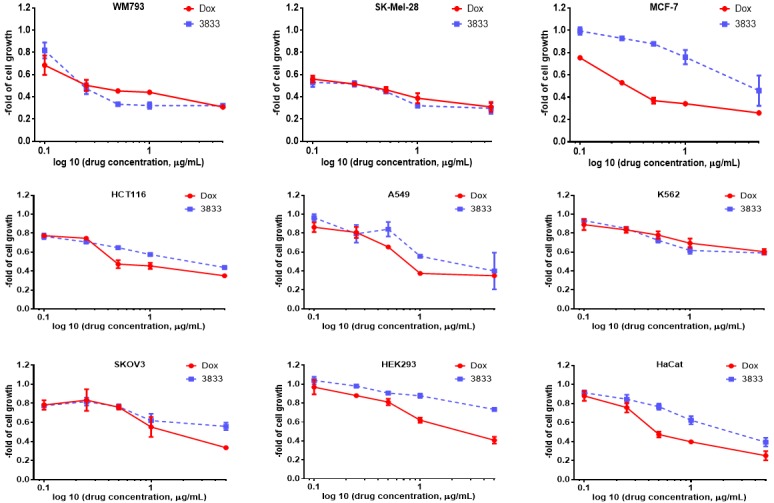
Growth inhibiting activity of Les-3833 and Dox toward different human cell lines. Cell vitality was estimated by the MTT assay after 72 h of experiment. Dox was used as a positive control.

*In vitro* screening of the cytotoxic action of Les-3833 and doxorubicin against different tumor cell lines was performed by MTT assay. The results of such testing of human breast adenocarcinoma MCF-7 cells, human lung adenocarcinoma A549 cells, human colon carcinoma HCT116 cells, human ovarian carcinoma SKOV3 cells are presented in Figure 2 and summarized in Table 1. The Les-3833 demonstrated very low activity against the ovarian carcinoma SKOV3 cells (half maximal inhibitory concentration (IC_50_) ˃ 5 μg/mL), and low activity against other human carcinoma cells of A549, HCT116, and MCF-7 lines (IC_50_ was 2.5 ± 0.19 μg/mL (A549 cells), 3.4 ± 0.32 μg/mL (HCT116 cells), and 4.5 ± 0.36 μg/mL (MCF-7 cells)). The cytotoxicity index (ie, IC_50_) of doxorubicin in all above noted cell lines was notably higher than of the Les-3833 (Figure 2, Table 2). An increase of the panel of tumor cells treated with the Les-3833 and doxorubicin by human myeloid leukemia K562 cells growing in suspension culture did not reveal a significant cytotoxic activity of either Les-3833 or doxorubicin (IC_50_˃5 μg/mL for both). The Les-3833 was less toxic (IC_50_ ˃ 5μg/mL) than the doxorubicin (IC_50_ = 3.2 ± 0.42 μg/mL) toward human embryonic kidney cells of HEK293 line and toward human keratinocytes of HaCat line (IC_50_ for Les-3833 was 3.16 ± 0.41 μg/mL and IC_50_ for doxorubicin was 0.47 ± 0.07 μg/mL, Figure 2, Table 1).

**Table 1:** Cytotoxicity (IC_50_) of Les-3833 and Dox toward different human cell lines (72 h, MTT assay)

Cell line	Les-3833 (ІС_50_±SD, µg/mL)	Dox (ІС_50_±SD, µg/mL)
Human melanoma WM793 cells	0.22 ± 0.03	0.24 ± 0.05
Human melanoma *SK*-*MEL*-*28* cells	0.3 ± 0.04	0.35 ± 0.06
Human lung carcinoma A549 cells	2.5 ± 0.19	0.8 ± 0.05
Human colon carcinoma HCT116 cells	3.4 ± 0.32	0.44 ± 0.09
Human breast adenocarcinoma MCF-7 *cells*	4.5 ± 0.36	0.3 ± 0.04
Human ovarian carcinoma *SKOV3 cells*	>5	2.0 ± 0.18
Human myeloid leukemia K562 cells	>5	>5
Human embryonic kidney HEK293 cells	>5	3.2 ± 0.42
Human keratinocytes HaCat cells	3.16 ± 0.41	0.47 ± 0.07

**Table 2:** Exposure time-dependence of Les-3833 and Dox cytotoxicity (IC_50_, 72 h, MTT assay)

Time (hours)	Les-3833 (ІС_50_±SD, µg/mL)	Dox (ІС_50_±SD, µg/mL)
6	>5	3.90 ± 0.55
24	4.20 ± 0.40	2.50 ± 0.43
48	0.85 ± 0.11	2.00 ± 0.38
72	0.22 ± 0.03	0.24 ± 0.05

The anticancer effect *in vitro* of the Les-3833 changed drastically when human melanoma cells were treated. It was found (Figure 2, Table 2) that the IC_50_ value equals 0.22 ± 0.03 μg/mL (WM793 cell line) and 0.3 ± 0.04 μg/mL (SK-Mel-28 cell line) which is more than 10 times lower than the IC_50_ noted above at the action of the Les-3833 toward various human carcinoma cell lines. This allowed to suggest a higher specificity of the cytotoxic action of the Les-3833 toward melanoma cells, comparing with that in human carcinoma cells. Doxorubicin possessed similar to the Les-3833 cytotoxic effect toward human melanoma cells of WM793 cells (IC_50_ = 0.24 μg/mL) and SK-Mel-28cells (0.35 μg/mL) (Figure 2, Table 1). It should be noted that the cytotoxic effect of the Les-3833 toward human melanoma WM793 cells demonstrated both dose-dependence (Figure 2) and time-dependence (Table 2).

### Cytotoxic action of Les-3833 toward human melanoma cells is accompanied by morphological changes in treated cells

**Figure 3:**
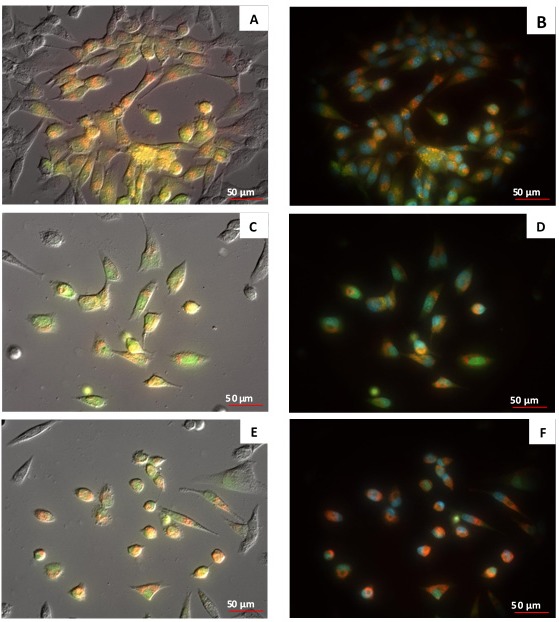
Human melanoma cells WM793 after 72 h treatment with the studied compounds: (**A**), (**B**) – control; (**C**), (**D**) – doxorubicin (positive control), (**E**), (**F**) – Les-3833, all used in 1 µg/mL dose. Left – DIC image of treated cells stained with Acridine orange. Right – fluorescent image of treated cells (blue color – staining with fluorescent DNA-specific dye Hoechst-33342, red and green color – staining with poly-specific fluorescent dye Acridine orange).

Following the cytotoxic action of the Les-3833 in comparison with the doxorubicin (Figure 2, Table 1), it was reasonable to estimate its cell damaging effect with the fluorescent microscopy. Treatment of human melanoma WM793 cells with the Les-3833 or doxorubicin (both used at 1 µg/mL) led to pro-apoptotic fragmentation of the nucleus, and chromatin condensation (Figure 1). Besides, most of the treated cells got a rounded shape. Red staining of cytosol regions with Acridine orange suggested the activation of the lysosomes caused by the Les-3833 in the melanoma cells. It should be noted that doxorubicin induced less visible damages in these cells (Figure 3).

### Les-3833 induces ROS production in human melanoma cells

**Figure 4:**
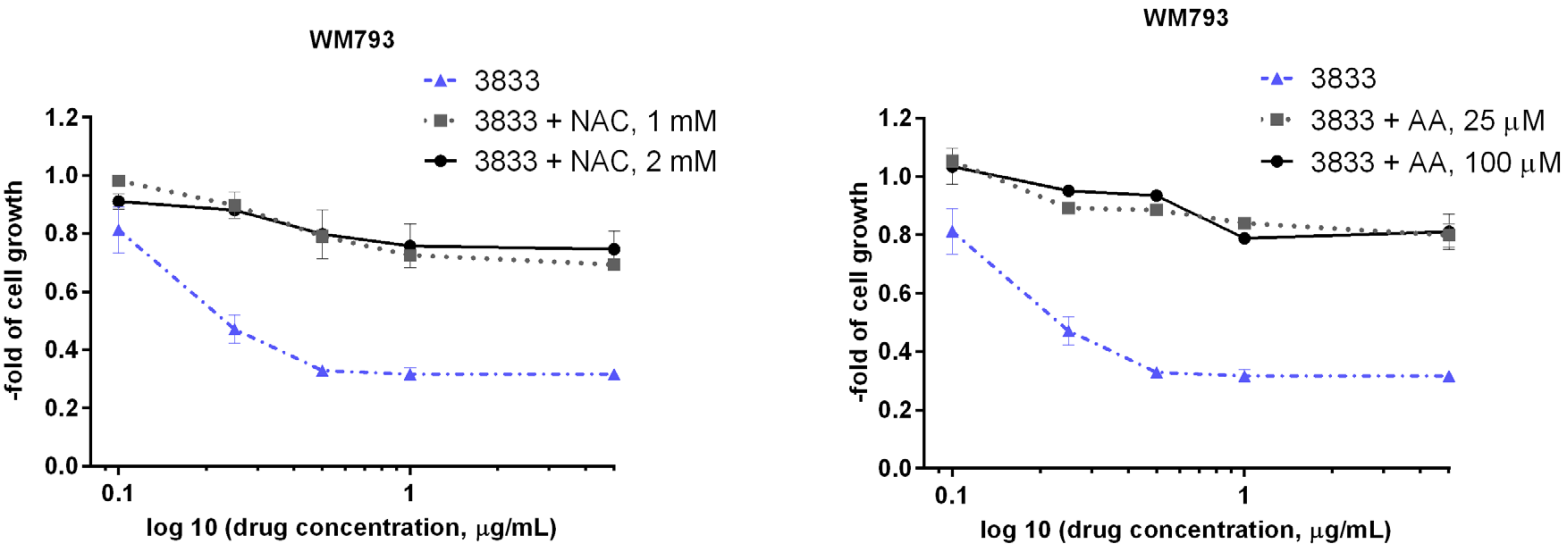
Protective effect of N-acetylcysteine (NAC) and ascorbic acid (AA) on the cytotoxic action of compound Les-3833 toward human melanoma WM793 cells. The Les-3833 was added to culture medium after 30 min pre-treatment of cells with NAC or AA. The viability of cells was determined by the MTT assay after 72 h effect of the compound.

It has been reported that several anticancer drugs induced a production of ROS that could be partly responsible for their cytotoxic action (17,18). In order to check whether the cytotoxicity of the Les-3833 relies on the intracellular ROS effects, we have used ROS scavengers such as NAC and AA. NAC is a precursor of glutathione and scavenger of H_2_O_2_ (14,18). The AA protects cells of the oxidative stress and it is a scavenger of the hydroxyl radicals (14,19). Both NAC and AA distinctly inhibited the action of Les-3833 and enhanced a survival of human melanoma WM793 cells (Figure 4). Thus, ROS production induced by the Les-3833 in melanoma cells might be one of the mechanisms responsible for its antineoplastic activity.

### Les-3833 induces an increase in ratio of Annexin V-positive melanoma cells

**Figure 5:**
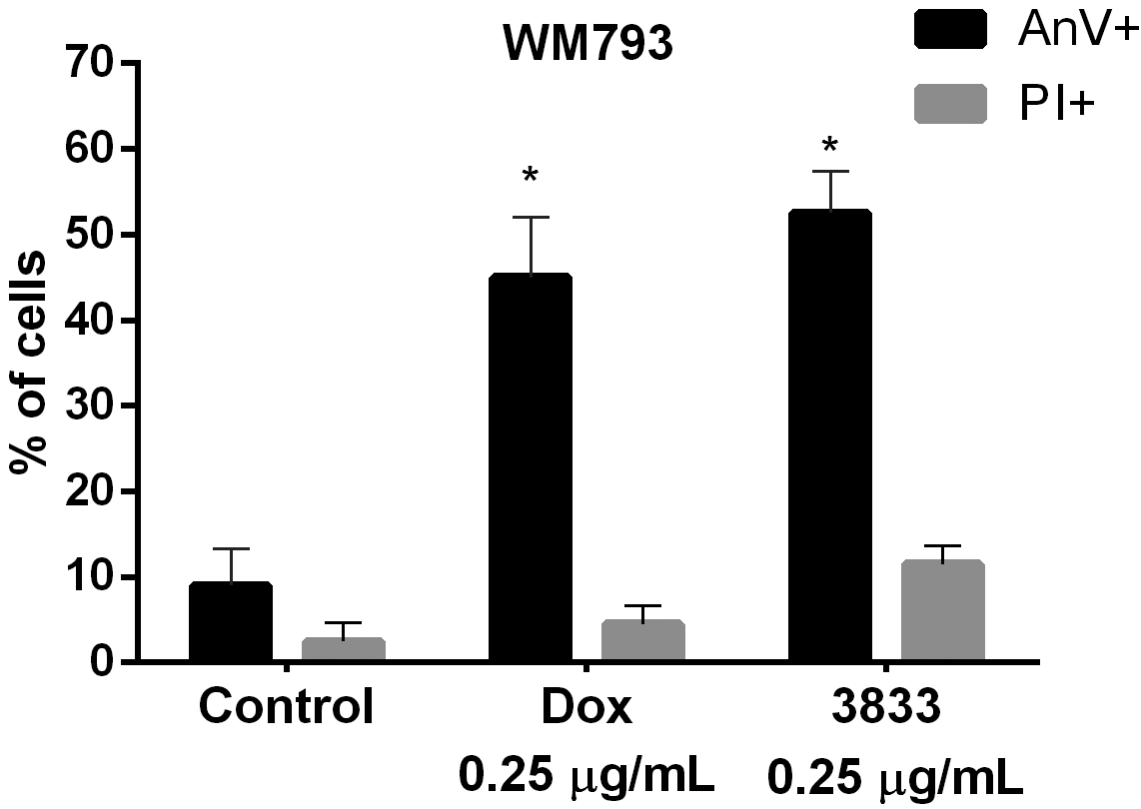
Effect of the Les-3833 and doxorubicin on induction of apoptosis in human melanoma WM793 cells. Ratio of the Annexin V/FITC positive (AnV+) and Propidium iodide positive (PI+) cells was analyzed by the fluorescent microscopy study of the Annexin V/PI double staining of WM793 cells treated for 72 h with the Les-3833 or doxorubicin both used in 0.25 µg/mL concentration. *P ≤ 0.05 (difference compared with the control).

Human melanoma WM793 cells were stained after treatment with FITC-conjugated Annexin V and PI, and then analyzed by fluorescent microscopy. The amount of the Annexin V-positive (apoptotic) cells affected by the Les-3833 (52.5%) was notable and higher than in control untreated cells (9.0%) (Figure 5). Under the action of the Les-3833, the amount of PI-positive (necrotic) cells increased (11.5%) in comparison to the control (2.5%), but much less than Annexin V-positive cells. These results suggest that the Les-3833 caused melanoma cell death mainly via apoptosis mechanisms.

### Expression of apoptosis-related proteins in human melanoma cells treated with Les-3833

**Figure 6:**
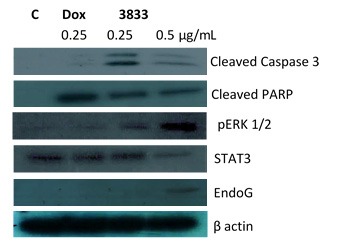
Pro-apoptotic proteins and transcription factor STAT3 (Western-blot analysis) in human melanoma WM793 cells treated for 72 h with the Les-3833. C – control (untreated cells).

To verify that apoptosis is induced by a specific agent, one needs a confirmation of apoptosis expression by using at least three alternative approaches. Thus, we have conducted the Western-blot analysis of the apoptosis-related proteins in the Les-3833-treated melanoma cells. The level of activated (cleaved) caspase 3 and inactivated (cleaved) poly-[ADP ribose]-polymerase-1 (PARP-1) was increased in melanoma cells treated for 72 h with the Les-3833 (Figure 6). Besides, compound stimulated phosphorylation of the extracellular-regulated kinase 1/2 (ERK1**/**2) belonging to the MAPK family; induced the EndoG proteins; and dose-dependently decreased the level of STAT3 (signal transducer and activator of transcription 3) protein (Figure 6).

### Changes in melanoma cell cycle pattern induced by Les-3833

**Figure 7:**
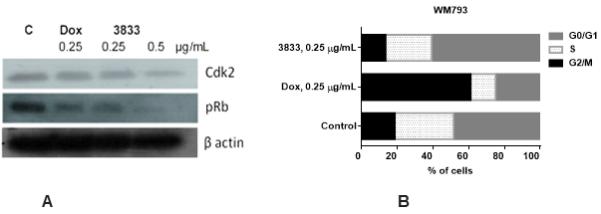
The results of measuring the effect of the Les-3833 (72 h treatment) on expression of cell cycle regulatory proteins and cell cycle distribution in human melanoma WM793 cells by using Western-blot analysis (**A**) or flow cytometry analysis (FACS) (**B**). C – control (untreated cells).

A loss of cell cycle control can be the indicator of cell transformation and carcinogenesis in the mammalians (20). We used the Western-blot analysis (Figure 7, A) and FACS analysis (Figure 7, B) in order to monitor changes in cell cycle-related proteins and changes in cell cycle pattern, correspondingly, under the action of the Les-3833 toward human melanoma WM793 cells. It was found that the treatment of the melanoma cells with this compound caused a decrease of cyclin-dependent kinase 2 (Cdk2) as well as a decrease of the phosphorylated Rb (Retinoblastoma) protein (Figure 7, A). These regulators are responsible for the G_1_ to S phase of cell cycle transition (14). The next step of our study was to use FACS analysis for monitoring the Les-3833-induced changes in distribution of melanoma cell cycle phases. We have shown that the Les-3833 caused a 12.3% increase (comparing with the untreated cells) of cell number in the G_0_/G_1_ phase, while the doxorubicin significantly increased (42.5% more than in the control) a cell number in the G_2_/M phase (Figure 7, B). These data suggest a principle difference in the molecular mechanisms of action of the Les-3833 compared with that of the doxorubicin.

**Figure 8:**
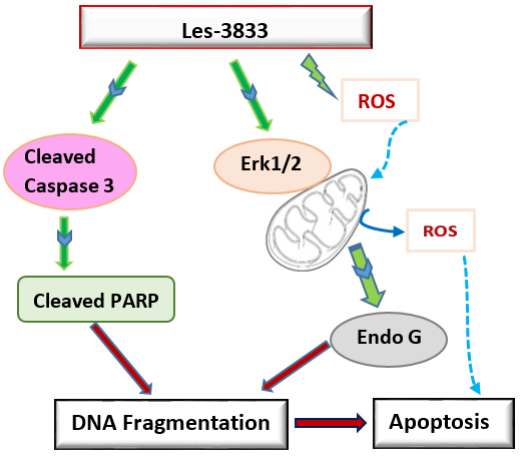
General scheme of signaling pathways induced by Les-3833 in melanoma cells.

Summarizing, 4-thiazolidinone derivative Les-3833 possesses high cytotoxic action against human melanoma cells of two different lines (SK-Mel-28 and WM793). Moreover, it is much less effective in inhibiting the viability of human carcinoma cells of various lines, and it has a very low activity toward human leukemia K562 cells, human embryonic kidney HEK293 cells and human keratinocytes of HaCat line. The Les-3833 induced apoptosis in human melanoma cells with an activation of caspase 3 and influenced the apoptosis-related proteins such as PARP, MAPKs, and Endo G. Probably, the Les-3833 affects the mitochondria-mediated apoptotic pathway (Figure 8). It increased a number of the Annexin V-positive melanoma cells and a number of such cells in the G_0_/G_1_ phase of cell cycle. In addition, the Les-3833 action led to the intracellular accumulation of ROS, since the cytotoxicity of this compound was inhibited by such ROS scavenges as N-acetylcysteine and ascorbic acid.

## Discussion

In our previous studies, cytotoxic action (MTT assay and Trypan blue exclusion test) of 3 structurally related 4-thiazolidinones – compounds Les-3833, Les-3882 and Les-3288 and Doxorubicin was compared for rat C6 glioma cells (21). It was revealed that melanoma cell lines were the most sensitive (IC_50_ in the MTT assay) to Les-3833, and that was the main reason for taking a decision to carry out more profound analysis of the molecular and cellular mechanisms of the cytotoxic action of this agent.

This study confirmed using broad panel of human tumor cells that the Les-3833 possesses the highest cytotoxic effect toward melanoma cells of WM793 line and SK-Mel-28 cell line. These effects were comparable with that of the doxorubicin. Thus, we have detected tumor tissue-specific toxic action of the Les-3833 for the melanoma cells, but not for other tumor cells of the epithelial origin. In case of mesenchyme-derived tumors Les-3833 had low activity against human myeloid leukemia K562 cells (IC_50_ ˃ 5 µg/mL), although in other studies a toxic action of this compound was shown for murine leukemia L1210 cells (IC_50_ = 1 µg/mL) (22).

Low cytotoxicity of the Les-3833 toward human embryonic kidney HEK293 cells and human keratinocytes HaCat cells should be stressed, since it is known that most anticancer drugs act not only on well proliferating tumor cells but also exhibit negative side effects toward the non-tumor cells (6). As predicted, the doxorubicin was highly toxic for all types of human tumor cells under this study, as well for human embryonic kidney HEK293 cells and human keratinocytes HaCat cells.

The revealed strong cytotoxic action of the Les-3833 toward human melanoma cells was confirmed by its damaging action on the morphology of these cells. It is known that specific pro-apoptotic changes in the morphology of the apoptotic cells can be observed in addition to biochemical changes such as DNA damage, activation of caspases, release of cytochrome C from mitochondria, or others (23). During microscopic studies of Les-3833 action on human melanoma WM793 cells chromatin condensation typical for apoptosis was found. Strong activation of lysosomes (Acridine orange staining of cytosol regions in red color) under Les-3833 treatment should be noted, since it was even higher than such activation induced by the doxorubicin. Acridine orange is a basic amino dye that possesses lysosomotropic action. After accumulation in lysosomes with acid pH, it demonstrates red fluorescence, while at neutral pH in the nuclei it emits yellow-green fluorescence. Uptake of foreign material by cells leads to activation of digestive vacuoles causing red fluorescence of accumulated Acridine orange in the lysosomes (24-26).

An increase in number of the Annexin V-positive melanoma cells at their treatment with the Les-3833 is strong argument in favor of the apoptotic way of death of these cells. It was shown that Les-3833 did not affect considerably a number of the Propidium iodide-positive melanoma cells that excluded a major role of necrosis during melanoma cell killing by this agent. The results of fluorescent microscopy measuring of the amount of the Annexin V- and the PI-positive melanoma cells under the action of the Les-3833 were comparable to doxorubicin, which indicated the anticancer potential of Les-3833.

In order to obtain more direct evidence of apoptosis induced by the Les-3833 in human melanoma WM793 cells, the activation of caspase 3 and cleavage of the reparation enzyme PARP-1 were monitored by the Western-blot analysis. We also found that ERK1/2/MAPKs, and EndoG levels were increased under the action of the Les-3833. Both caspase 3 and PARP-1 belong to principal biochemical markers of apoptosis (27), while the protein kinases ERK1/2/MAPKs were shown to respond the action of various stressing agents, including the anticancer drugs (5,6). PARP-1 used NAD^+^ to cause the poly(ADP-ribosyl)ation of proteins (27). A design of new anticancer chemotherapeutics is intensively developing for creation of novel PARP inhibitors. Thus, the intactness of PARP-1 and PARP-2 could be the prognostic biomarkers at drug development (5,6,27). PARP-1 degradation induced by the Les-3833 in human melanoma cells demonstrates an additional mechanism in the biological action of this potential anticancer drug.

Similar compounds - troglitazones and 3-(2-amino-ethyl)-5-(4-ethoxybenzylidene)-thiazolidine-2,4-dione - induced the inhibition of growth of human pancreatic cancer cells and human leukemia cells through the MAPK signaling pathway (28-30). It was shown that 5-ene-4-thiazolidinones induced apoptosis in the acute promyelocytic leukemia HL-60 cells via modulating MAPK signaling pathway (14). Thus, the activation of MAPK/Erk1/2 pathway in tumor cells might be a common mechanism of cytotoxic action of 4-thiazolidinones, including the Les-3833, toward human tumor cells.

It was reported that the mechanisms of anticancer effect of 4-thiazolidinones and similar heterocycles could be related to the activation of caspase cascade, changes in the intracellular Ca^2+^ and mitochondria functioning, a release of cytochrome C from mitochondria, and/or the endoplasmic reticulum stress (6,28). EndoG (endonuclease G) is a mitochondrial protein released at apoptosis, and it is involved in caspase-independent DNA degradation (31). EndoG elevation induced by the Les-3833 in melanoma cells allow one to suggest that these cells undergo apoptosis via the mitochondria-mediated pathway. Interestingly, the doxorubicin did not affect the level of the EndoG protein in treated melanoma cells that suggested different mechanism of action of Les-3833 and doxorubicin in these tumor cells.

STAT3 is a transcription factor involved in regulation of expression of different genes under the action of various growth factors and cytokines that influence cell growth and apoptosis (5,6). STAT3 level was decreased in human melanoma WM793 cells treated with the Les-3833 for 72 h, while the doxorubicin did not affect the level of this transcription factor. Thus, the action of Les-3833 is mediated via various signaling pathways in the cell suggesting a uniqueness of this potent anticancer substance. Les-3833 induced apoptosis in human melanoma cells with an activation of caspase 3 and influenced the apoptosis-related proteins such as PARP, MAPKs, and Endo G. Probably, the Les-3833 affected the mitochondria-mediated apoptotic pathway (Figure 8).

It was reasonable to compare the action of the Les-3833 with that of the doxorubicin on cell cycling and the levels of the related regulatory proteins. We have found that Les-3833 decreased the level of both Cdk2 and phosphorylated form of Rb protein in human melanoma cells of WM793 line. The activity of cyclin-dependent kinase 2 is important for the G_1_/S transition, and the retinoblastoma protein is a tumor suppressor whose action is impaired in many tumors. Inactivation of the Rb protein by phosphorylation runs the cell cycle progression (5,32,33). Rb phosphorylation in the G_1_ phase is catalyzed by the CDK2/Cyclin E complex, and it is needed for cell transition to the S phase (14,33). The Les-3833 and doxorubicin induced similar degree in decrease in both the Cdk2 and Rb proteins, however, the distribution of cell cycle phases under the action of these drugs differed significantly. While doxorubicin, as predicted (14,34), arrested melanoma cells in the G_2_/M phase, the Les-3833 induced the accumulation of these cells in the G_0_/G_1_ phase. The arrest of tumor cell cycling in either G_0_/G_1_ or G_2_/M stages is of great interest, and it strongly depends on the expression of cyclin-CDK inhibitors - p21 and p27 proteins (32).

ROS are important in the action of several anticancer drugs, however, these agents are also damaging cells of normal tissues and organs (negative side effects) (14,17,18,21,35,36). The results of our studies suggest that the action of the Les-3833 in human melanoma WM793 cells is dependent on ROS induced by this agent, since NAC and AA blocked the cytotoxic effect of the Les-3833. It was reported that doxorubicin disturbed a balance of ROS and enzymes of the antioxidant defense in blood serum of laboratory rats that could be one of the reasons of high general toxicity of this anticancer drug. At the same time, 4-thiazolidinones derivatives Les-3288, Les-3833, and Les-3882 were much less effective there (21). The level of the malonic dialdehyde (indicator of ROS content) was also increased at the Les-3833 action, although a magnitude of that increase was less than at the action of doxorubicin (21). Probably, in the cytotoxic action of the Les-3833, ROS play more important role than they play it in the action of other 4-thiazolidinones derivatives (Les-3288 and Les-3882). In a recent study, we have demonstrated that 5-[5-(2-Hydroxyphenyl)-3-phenyl-4,5-dihydropyrazol-1-ylmethylene]-3-(3-acetoxyphenyl)-2-thioxothiazolidin-4-one that is 5-ene-4-thiazolidinone derivative was highly toxic for human promyelocytic leukemia cells of HL-60 line (IC_50_ = 118 nM) with low toxicity toward human embryonic kidney cells of HEK293T line and murine mesenchymal preosteoblastic cells of KS 483 line (14). This potent drug caused mitochondria-depended apoptosis and induced G0/G1 arrest in treated cells. It also activated ROS production (14).

Other investigators found that ROS level was increased by 5-ene-4-thiazolidinones in human colorectal adenocarcinoma HT29 cells and the acute lymphoblastic leukemia CEM cells (18). In addition, the action of many chemotherapeutic agents is based on increasing ROS levels, and the resulting induction of the irreparable apoptosis-related damages in tumor cells (17). ROS-induced apoptosis can be linked to the mitochondrial oxidative stress causing cytochrome C release from mitochondria and activation of caspases (36).

### Conclusion

Toxic action of Les-3833, a novel 4-thiazolidinone derivative, toward 7 human tumor cell lines was studied, and human melanoma cells of WM793 and SK-Mel-28 lines were found to be the most sensitive. Human carcinoma cells of various tissue origin, as well as human embryonic kidney cells of HEK293 line and human keratinocytes of HaCat line, were relatively resistant to such action. Results of Annexin V/PI staining of melanoma cells, character of activation of caspase 3, PARP, MAPK, and EndoG protein, and the morphological changes suggest induction of apoptosis in Les-3833-treated cells. This agent also induced ROS production in melanoma cells and their arrest in G_0_/G_1_ phase of cell cycle.
